# Lessons from the Conservatory Model as a Basis for Undergraduate Education and the Development of Intelligence

**DOI:** 10.3390/jintelligence10020034

**Published:** 2022-06-15

**Authors:** Robert J. Sternberg, Linda Jarvin, Ophélie Allyssa Desmet

**Affiliations:** 1 Department of Psychology, College of Human Ecology, Cornell University, Ithaca, NY 14853, USA; 2 Paris College of Art, 75010 Paris, France; lindajarvin@gmail.com; 3 Department of Human Services, Valdosta State University, Valdosta, GA 31698, USA; odesmet@valdosta.edu

**Keywords:** conservatory, liberal arts, musical performance, entrepreneurship, problem solving, adaptive intelligence

## Abstract

We review the musical conservatory as a model for educators to learn how to enhance admissions, instruction, and assessment in liberal arts collegiate settings. Although conservatories serve primarily students wishing to enter musical careers of various kinds, the model on which they are based can, in many ways, serve any student and any school. We review some of the history of conservatories and describe how they work. Next, we explore how they develop a wide range of technical, cognitive, affective, and conative skills. Finally, we show how the skills they develop are important not just for music students but also for all students who will enter the world of work and face difficult and unexpected adaptive challenges.

## 1. Introduction

Future musicians may choose between a traditional undergraduate liberal arts education and an education in a musical conservatory. The two models are rather different and focus on somewhat different knowledge and skills. The liberal arts model provides more of a general education, whereas the conservatory model is seen as providing a more strictly preprofessional education. However, the differences may not be quite what they appear to be, and an ideal education for any student might combine elements of each.

In this article, we discuss whether a blend of the liberal arts and conservatory models might have advantages not only for musicians but also for all students who wish to prepare for the adaptive challenges of the mid-21st century. One key advantage of the conservatory model is to explicitly aim to develop not only students’ academic skills, but also their psychosocial skills as they apply to real-world careers and related adaptive contexts. In contrast, the traditional liberal arts model is primarily concerned with academic and cognitive skills. We even live in a time when some states in the United States are trying to eliminate prosocial skills, even at the elementary level where they are usually taught, using the argument that such skills represent a hidden “political” agenda (Goldstein and Saul 2022).

In liberal arts education, at least in the United States, students generally engage in a variety of courses designed to teach them basic knowledge about the world in the first two years of college. They then specialize in a major field of study in the last two years (Sternberg 2016). Sometimes, there are required courses, or required choices from lists of prespecified courses. Generally, the idea is that students will learn basic knowledge about, and skills for coping with the world in the first two years, and then the subject matter that will serve as a basis for specialization in the second two years. Under this model, even an engineering student might take courses in literature, philosophy, or the arts during the first two years, and then move on to the specialized concentration in engineering in the latter half of their college career. This liberal arts approach to higher education, with an emphasis on breadth of academic courses early on, is gaining traction in other countries, including Europe (van der Wende 2017). Such a broadening of the liberal arts approach makes sense, because technical information changes, but the broad thinking skills one needs to adapt remain relatively constant, although their emphases may change. For example, recognizing conspiracy theories and distinguishing disinformation from true information are skills that have become especially important in recent times (Sternberg forthcoming).

Although the liberal arts approach sets out to develop a broader range of skills than a professional education does, we argue that it does not, in its current form, sufficiently focus on building the cognitive, emotional, and social skills that are needed to deal with the adaptive challenges of the world of the 21st century.

The conservatory model also typically provides liberal arts education but in a different context. For example, consider a student studying to be a brass player (trumpet, horn, tuba, trombone, euphonium) at the New England Conservatory of Music. The future musician will take liberal arts and modern-language courses, but also studio, brass, music theory, music history, entrepreneurial musicianship, ensemble, chamber music, and recital courses (https://necmusic.edu/sites/default/files/documents/2021-2022%20Academic%20Catalog.pdf (accessed on 10 June 2022)). Specialization in other areas will yield a similar course program geared toward the other specialty, such as strings, voice, or composition.

We believe that the conservatory model offers learning-related and general-intelligence-related skills beyond those provided by the typical liberal arts curriculum. What particular skills does the conservatory model offer for all students, not just for students of music?

## 2. Basis in the Theory of Adaptive Intelligence

Using the theory of adaptive intelligence (Sternberg 2021a) as a theoretical framework, we explore several skills central to the conservatory model that would benefit all students. Adaptive intelligence comprises a collection of skills, attitudes, and behaviors based upon creative, analytical, practical, and wise thinking (Sternberg 2021a). The goal of adaptive intelligence is, as its name implies, a broad adaptation to a given environment. Broad adaptation encompasses narrower adaptation, which is modifying oneself to fit an environment. However, it also encompasses shaping an environment to provide a better fit of the environment to oneself, and selection of environments to account for the fact that, sometimes, the environment in which one finds oneself is a bad fit or even a potentially deadly fit, as in much of Ukraine at the time we are writing in mid-2022 (e.g., Murphy 2022).

We suggest that the conservatory model successfully identifies and promotes adaptive intelligence. Furthermore, we believe that all students would benefit from developing their adaptive intelligence through aspects of the conservatory model. As such, there are several lessons that liberal arts programs could carry over from the conservatory model. These are lessons that can be learned from adaptive intelligence as applied directly to the domain of musical composition and performance (Sternberg 2020c). 

Another related take on musical intelligence is that of Gardner (1983, 2011). Gardner views musical intelligence as one of eight multiple intelligences, each independent of the others. Musical intelligence involves skills needed for playing a musical instrument, composing, conducting, singing, dancing, and other musical activities. The other seven intelligences in Gardner’s theory are linguistic, logical–mathematical, spatial, naturalist, bodily–kinesthetic, interpersonal, and intrapersonal.

In Sternberg’s (2020c) theory, to succeed as a professional musician, one first needs to be highly analytical. One needs to understand what musical messages the composer intended to convey, how they are conveying the messages, and how those messages can be translated into instrumental (or voice) music. For example, what particular actions of the hands or breath (or voice) can achieve the desired effects? How can these effects be made to be more effective?

Memorizing pieces may be part of one’s task; however, as in the liberal arts education, mere memorization does not prepare one for the challenges one will face in one’s life and career. Moreover, and importantly, one must be able to analyze one’s own performance so that one can practice deliberately to improve one’s performance. Simply playing the music repeatedly will not improve one’s performance much, if at all. Mindless repetition is not a viable path to expertise (Ericsson and Pool 2017; Sternberg and Kibelsbeck 2021). In the same way, simply memorizing the material in a textbook will not improve one’s ability to cope with life and career. In life and career, one must constantly analyze problems and one’s own processes of solution, seeking to improve one’s ability to face the challenges the world presents. Jarvin and Subotnik (2010) interviewed gatekeepers in classical music (artistic directors, newspaper critics, talent managers) about the factors that they perceive to be most important to a performer’s success at different stages of their development, from student to internationally acclaimed professional. Respondents identified three key analytical skills: ability to analyze music, knowing one’s strengths and weaknesses, and pertinent risk taking. The ability to analyze music is especially important in the early stages, when a performer must refine their understanding of the patterns and structures in music so that, when they enter a professional career, they have mastered the ability to learn new material quickly. As students grow older, though, it is important for them to analyze their own strengths and weaknesses to develop the necessary self-knowledge to capitalize on strengths and make the right choices for the future. Additionally, for the professional, risk taking is perceived as a sign of artistry. It requires an analysis of the available options to take those risks that will prove rewarding.

Second, to succeed as a professional musician, one needs to be technically proficient and also be *creative* in one’s interpretation of the music (Subotnik et al. 2003). Only creative performers can imagine different options and take the appropriate risks discussed above. Child prodigies may be unusually technically proficient, but they cannot succeed as professional musicians unless they go beyond technical proficiency. Their musical interpretation must be (a) novel—distinguishing them from other musicians; (b) appropriate—it matches the music and its period; and (c) effective—it connects with its audience. In contrast, many undergraduate programs place relatively little emphasis on creativity, and even fewer assess students’ creative potential. Yet, in the mid-21st century, creativity will matter in practically any professional or other life pursuits: The ways of the past are not adequate when the world changes at such an astonishing rate, and when we need to equip students with creative problem-solving skills (Grant 2017; Kaufman and Sternberg 2019, 2021; see Plucker 2016).

Third, to succeed as a professional musician, especially in current and predicted near-future times, one needs to develop a vast repertoire of practical skills. Practical skills encompass emotional skills such as persistence and restoring self-confidence after a setback, and social skills such as collegiality and effective and appropriate self-promotion (Subotnik et al. 2016). How does one prepare for and perform effectively in an audition? How can one be entrepreneurial in creating new forms of musical performance and collaboration and in reaching existing and new audiences? How can one succeed when conventional audiences are sparse (as in the times of COVID-19)? If one seeks a career as a soloist, then how can one project a kind of charisma that singles one out from the pack? If one seeks a career as an orchestral, band, or chamber player, then how can one collaborate with others to produce music that is more than just the sum of its separate parts—that harmonizes and synergizes with other members of the group? How can one teach these skills to younger people, some but not all of whom will become professional musicians? Similarly, in any career, one needs to develop the practical skills needed to survive in one’s life and in one’s occupation—the tacit knowledge of one’s professional success (Sternberg 1994). Too often, students graduate from college without these skills, thereby slowing down their integration into the professional world. Students who have not acquired these practical skills in their home environment (and have not been provided them in college) will be at a particular disadvantage. If they do not quickly pick these skills up on the job, they fall behind or potentially find themselves jobless.

Fourth, good conservatory training will also develop in musicians the *wisdom* to make the world a better place. This wisdom is seen in a variety of ways—the organization of benefit concerts to help charitable causes, the performance of music to groups in hospitals and schools and homes for the aged who need spiritual sustenance, the commissioning of music from struggling (and not-so-struggling) composers, playing music to calm and distract an audience that is sheltering from bombs (https://www.themoscowtimes.com/2022/03/17/violin-becomes-weapon-of-resistance-in-ukraine-shelters-a76981 (accessed on 10 June 2022)) or in-between air raids (https://www.thestrad.com/news/solo-cellist-performs-in-central-kharkiv/14642.article?utm_source=adestra&utm_term=&utm_medium=email&utm_campaign=25628 (accessed on 10 June 2022)), and the communication to audiences that music is a common language that everyone can speak, even if there are somewhat different “dialects” through which it is conveyed. 

The original meaning of the “conservatory” was as a place for orphans to find refuge and be educated in the shared language of music—a wise way to encourage social cohesion. Great musicians are wise musicians. Similarly, great professionals in any field are wise as well (Glück and Weststrate forthcoming; Grossmann et al. 2020; Jeste and LaFee 2020; Schwartz and Sharpe 2011). They give back to the world—they help to achieve some kind of common good that goes beyond their own resources or reputation (Sternberg and Glück 2022a, 2022b). 

Fifth, musicians have different patterns of talents. No two musicians have exactly the same pattern of gifts and talents. A successful musician figures out what they do well and what they do not do as well, and then develops, as possible, a repertoire and techniques that enable them to show what they do particularly well. However, the same principle applies in all fields. Writers, artists, scientists, teachers, nurses, businesspeople, and everyone else have unique patterns of strengths and weaknesses. They succeed in their careers to the extent they can capitalize on their strengths and correct or compensate for their weaknesses (Sternberg 2020c). They show what their unique contribution to the world can be. In the conservatory model of education, this is acknowledged, and students are guided toward developing the best version of themselves that they can be. Would not all students benefit from such a pursuit?

Finally, successful conservatory training helps develop a *passion* for music as a way to make the world not only a different place but also a better place. Music can help bring the world together. It is universal. The world is so fraught with problems today—violence, climate change, pollution, poverty, dictatorships, and the like. If there has ever been a time when we need to focus on not just individual but also species wellbeing and survival, this is one such time. In any field, people need to leverage their skills in a positive and transformational way, not in a way that merely makes some individual or group a profit. 

There are three basic differences, we would argue, that typically differentiate conservatory from liberal arts education:

1. *The conservatory has as its fundamental mission to preserve.* The word “conservatory” comes from the Latin word conservare, which means “to preserve.” To elaborate on earlier statements, the Italian conservatorio was originally designated an orphanage or hospice that took in orphans and initiated them to music: The first known conservatorio was founded in Naples in 1587 (*Santa Maria di Loreto*, founded by Giovanni di Tapia). Antonio Vivaldi served as the music director of the *Conservatorio de la Pietà* in Venice (Larousse 2005). One could argue over what a conservatory preserves: the culture of a society or of civilization, musical compositions, forms and traditions, talents, etc. In its early mission, to provide orphans with musical skills, it also provided children with a language that would allow them to integrate into the society and culture into which they were born. Regardless of what exactly is preserved, we will argue that this fundamental mission of conservation matters a lot, in the long run.
2. *There is an ultimate performance criterion.* The conservatory student has an ultimate goal—performance. They will perform on an instrument, or they will sing, or they will compose and hear their composition performed, or they will teach. However, in each case, there is a kind of performance on the basis of which they ultimately will be judged. In liberal arts education, there is typically a collection of courses, but none of them really matters in its own right to one’s future career. Although accreditation agencies have pushed for more culminating capstone experiences for graduating seniors (Gray and Schermer 2011), at least in the United States, most Bachelors’ programs somehow do not culminate in an ultimate performance criterion that matters in its own right. A grade-point average (GPA) is simply an average of course performance—it is not a distinctive performance in its own right.
3. *The various strands of knowledge and skills need to be merged into the capstone performance.* Although students take a variety of courses in a conservatory—as they do in a liberal arts college program—because the conservatory has the ultimate performance criterion, the various aspects taught need somehow to be synthesized into a successful performance. In college, one can have isolated knowledge bases for all the different courses one takes, and it may not matter. In a conservatory, that will not work—if one cannot somehow synthesize them into successful performance, they will not matter. In that sense, the conservatory model not only encourages transdisciplinarity, but also makes the combination of learning-related skills a requirement for success.

Conservatories explicitly, then, do what all undergraduate institutions need to do. They prepare their students for the world the students will encounter, not just for a hypothetical world of multiple-choice problems with quick solutions and seemingly “correct” answers (Sternberg 2020b). All educational institutions could learn from conservatories how to provide a better education—to create more adaptively intelligent individuals—that prepares students for the world of the future.

Any form of education, including the conservatory form, has limitations that need to be acknowledged as well. What might some of these limitations be for the conservatory model? Consider three potential limitations. 

First, although conservatories typically offer liberal arts options, these options are usually limited. The emphasis is on disciplinary rather than interdisciplinary education, although there might be interdisciplinarity within the world of music (e.g., instrumental music and voice). Additionally, music—or at least the theory of music—has a certain structure that not all disciplines have. The model is more similar to the European model of education, where students choose a specialization right from the beginning of their undergraduate years. In Germany, for example, one applies for a particular field of specialization in the undergraduate program of the university, and admission is by specialization. 

Second, a conservatory has the upside that, if a student wants to enter a musical career, employers know that the student is very well prepared for a career in music. However, if the student decides on a career in another profession or decides for advanced training in another field, decision makers may view the student as too narrowly educated to be worthy of the risk in selecting them.

Third, styles in all fields change over time. For example, the best players of the Bach Six Suites for Cello of the 20th century, such as Mstislav Rostropovich, sound quite different from the best players a generation later, such as Yo-Yo Ma. Similarly, Fritz Kreisler’s romantic style of playing the violin is unlikely to be mistaken for a predominant style in 2022. Part of what a liberal arts education prepares one for, at least in theory, is the fact that there is little stability in life—whatever preparations one makes will last only so long.

These limitations are only potential. Any conservatory can, in theory, offer any liberal arts courses it wishes to. Employers and graduate schools can look at transcripts and other records and realize that, just because a school follows a conservatory model, it does not mean the education that it offers is narrow. Additionally, students can be prepared for changing trends in any educational environment—liberal arts, conservatory, or otherwise. These are challenges, but not insurmountable ones. In the end, the best preparation for life, we believe, combines aspects of conservatory education with aspects of liberal arts education.

## 3. The Special Functions of Conservatory Education Elaborated

The crucial aspect of conservatory education, we would argue, is in the name—conservatory. The education is based on conservation. We suggest that such a notion would apply particularly well to the world today because there is such a pressing need to conserve—in the traditional sense of conserving natural resources, of course, but also of conserving civilization as we know it. Civilization is under so many threats today—dictators massacring people and, in one case, destroying a country, to feed their egos and their fantastical notions about their countries’ historical place in the world; a pandemic that has killed over six million people as of March, 2022 (https://www.who.int/emergencies/diseases/novel-coronavirus-2019 (accessed on 10 June 2022)); the devastation of natural resources, in part because of irresponsible leadership in countries with leaders who do not care about the environment they leave to future generations; global climate change, which is progressively making the world less and less livable not only for humans but also for millions of other species; extreme poverty and famine killing people while billionaires take luxury cruises into outer space. We need to use our intelligence to advance not only our own selfish interests, but the conservation of the world for our own species and for many others (Sternberg 2021b). 

The other crucial aspect is the integration of knowledge and skills. Performance on a musical instrument (including the human voice), in composition, or directing, is the ultimate test of success in conservatory education. In music, one will be judged not on one’s independent bits of knowledge or assorted isolated skills, but on how one can integrate one’s knowledge and skills into performance. This integration is often lost in traditional college education. One often is judged in college on little more than one’s coursework and perhaps a GPA averaging grades in that coursework. Even if there is a “senior project” or capstone, it rarely fully integrates the knowledge and skills one has acquired during one’s college education. Conservatory education is devoted to the solution of a problem: How can I perform in a way that will display my musical excellence and set me apart from others who may be technically proficient but who lack the particular unique creativity, analysis, practical skills, and wisdom I can bring to my profession? These questions promote lifelong learning, as they will be answered differently throughout a performer’s development. We suggest that a college education in a liberal arts tradition might do the same.

Conservatories have one other special feature. Students are continually evaluated and compared not only to each other, but also to absolute standards of excellence in musical performance. In contrast, it is possible, in many liberal arts programs today, to just get by. Because of grade inflation, just getting by may result in grades that are respectable and even high. Sternberg (2016) has discussed trends in current education that lead to “mediocracy”, or the valuing of a mediocre performance as good enough, or even, in some circumstances, as excellent (such as when students are evaluated for adherence to some particular dogma) (see also Hermanowicz 2013). There is much to be said for a curriculum whose very structure ensures a certain level of excellence. 

## 4. Applying the Conservatory Model

In musical training, one always keeps in mind that one is training to make a difference to a musical audience. In the theory of adaptive intelligence, the audience is humanity—not just humanity of the present but also of the future—our children and our children’s children, onward into the future. 

As in musical training, one approaches the problem of doing what is best for one’s audience in a multidisciplinary way. For example, in musical performance one must have the technique to play pieces successfully, but also creative, analytical, practical, and perhaps wisdom-based skills. How might this “play out” for solving a problem for one’s current and future audience, such as global climate change? For one thing, one is taking a problem-based approach, as one would, say, if one wished to provide the best rendition of the Bach Six Suites for Cello. 

First, just as one learns in a conservatory musical theory, in solving the problem of global climate change, one would need to understand the theory underlying climate change and why it is happening. How and why do greenhouse gases accumulate? How does the accumulation of these gases lead to changes in the climate? What kinds of chemical and meteorological changes take place in response to them (see IPCC 2022)? 

Second, in a conservatory, one learns music history. To understand the phenomenon of global climate change, one needs to understand its history and how it has come to be so problematic for the world. How have the changes accumulated over time? Why are they so much more severe in our lifetime than in much of the past? Is the history a continuous one, or do the changes become catastrophic after reaching a tipping point? What approaches have been tried in response to climate change, and how were they effective, or not?

Third, in a conservatory, one learns about playing in ensembles, chamber music groups, orchestras, and/or bands. That is, one learns to solve musical problems in groups of different sizes and composed of individuals with different experiences and areas of competency. Real problems of consequence are almost always solved in groups, not individually. Problem solvers with different kinds of expertise (as in an ensemble) work together toward a common goal. Often, one can get through a college education without having learned to work with others. Even in the instances when group projects are assigned, it is rare that students are explicitly taught to work effectively in teams; most of the time, those students with prior leadership skills take the lead, rather than all participants learning about project management and effective governance. The result is graduates, such as many of our politicians, who become show-boaters. They cannot work with others but only for their own self-advancement, with a thin veneer of offering or pretending to help their constituents. To solve a problem such as climate change, people will need to work together, as professionals—climatologists, meteorologists, chemists, biologists, and of course, businesspeople and laypeople—toward the common goal of producing a climate that is more conducive to quality of human life and the lives of other species (Berry et al. 2018; Mahaffy et al. 2021).

Fourth, in a conservatory, students study musical entrepreneurship, which has become increasingly important, especially in the age of COVID-19. Students learn how to build a business, whether a social business (Yunus 2013) or a profit-driven one that showcases their talents, individually or collectively. It has become clear that the problem of global climate change will not be solved without entrepreneurs of many kinds offering products and services that are alternatives to those of the big oil, gas, automotive, and other companies that have large investments in currently used fossil fuel fields.

Fifth, conservatory students develop and practice conative and motivation-related skills and attitudes to help them persist through the highs and lows that accompany a musical career. Pursuing a musical career is notoriously difficult because of the highly competitive nature of the field. Therefore, being technically proficient is not at all sufficient to navigate a professional musical career successfully. Successful artists are self-confident, persistent, passionate, and intrinsically motivated about their music (Jarvin 2017; Subotnik et al. 2003). They possess a willingness to take risks and to continuously engage in self-assessment for self-improvement (Subotnik et al. 2003). Therefore, students are explicitly taught conative and motivation-related skills. Although not all talent domains are as competitive as the musical arts, all students, regardless of their chosen career, would benefit from explicit instruction and practice of conative skills and attitudes. Learning to persist and assess your own strengths and weaknesses, for example, will set students up to be lifelong learners in any field. 

Sixth, and perhaps most important, conservatory students know they will be judged, in the end, on their performance, whether in a studio or as a soloist, or as a member of a group. The “grade” is not what matters. What matters is whether they have the knowledge and skills effectively packaged for action. This, too, has been missing from a college education, where actual activities often take the form, at best, of extracurricular activities. Students need, whether through internships, service learning, or cooperative education, to get out and deploy the knowledge and skills they have learned in the classroom. Climate change, like all complex issues, requires students to engage in systems thinking and understand how the different components of the issues interplay and impact one another. In music, technical wizards who cannot play in harmony with others in the orchestra will not produce a great performance, and neither will a collegial quartet where each individual lacks technical skill or emotional depth in their interpretation. When it comes to climate change, this complexity can be experienced by participating in an activity such as constructing a mural showing how the factors identified in the 2022 Intergovernmental Panel on Climate Change report interact. In music, this complexity is experienced in attending a performance, which brings together the technical skills of the interpreters, the harmony of their interaction with each other, and the creativity expressed in the interpretation of the score.

## 5. Admissions

Admission is a way for an institution to signal what is important to them. What are they looking for, and what are they trying to develop? Who is considered suitable to study in their institution? A school that counts almost exclusively on mathematics and science performance would send a very different message from that of a school that looks more broadly. Admission to an undergraduate program can be either open or competitive. In conservatories and most liberal arts schools, admission is at least somewhat competitive. However, the criteria are quite different. 

In a conservatory, the ultimate goal is to produce musicians of the first order. The best way to predict future musicianship is to assess current musicianship. This assessment usually is carried out through an audition of some kind, typically in person but, especially in the times of COVID-19, possibly online or by other means that do not require in-person performance. The performance matters a lot. Someone may have achieved straight A’s in high school or high standardized test scores, but those are not going to help much in the prediction of musical success. Even grades in music courses will not predict success well because conservatories are more competitive than typical high schools. Even music programs in traditional liberal arts schools are typically much more competitive. The ultimate goal of a conservatory is to produce accomplished musicians, so the audition of musical performance will matter a great deal (Subotnik and Jarvin 2005) because it is probably the best indicator, at least prior to entry, of how candidates will fare as performers. 

Traditionally, in competitive conservatories, the audition is a one-shot opportunity to play a predetermined repertoire. It is a highly stressful event, and the experience mimics the realities of a performance in front of an audience (Jarvin 2017). Another key feature of the audition process is that it can be carried out behind a screen, meaning that the committee rating the audition performance cannot see the candidate (Bennett 2008). The rationale is that the assessment of the performance will thus be less impacted by the candidate’s visible and physical attributes, creating a more equal playing field. 

Additionally, conservatory admissions procedures focus on identifying students’ teachability and potential (Jarvin 2017). Music education and higher levels of talent development in any field require a level of individualized learning trajectories. To accommodate those individual learning trajectories, conservatory teachers implement ongoing, formative assessments focused on the students’ responsiveness to the curriculum and instruction. This teaching philosophy is present in the admissions procedures as well. Many conservatory programs offer students who have demonstrated great potential, but lack readiness or technical proficiency, the opportunity to participate in a preparation year. This preparation year allows students to receive training and support to best prepare them for successful participation in the conservatory program. Conservatory educators have realized that conative aspects such as passion, intrinsic motivation, persistence, self-confidence, and risk taking can make up, to some extent, for limited technical proficiency (Jarvin 2017). Thus, the admissions procedures are designed to assess not only technical proficiency, but also teachability and the aforementioned conative factors.

Liberal arts programs do not have a comparable means of assessment. Students may submit grades from high school, standardized test scores, the Common App, or some other application answers and essays, letters of recommendation, and perhaps even some kind of work they have done. Many of these (e.g., name of high school, experience described in the personal statement, profession of the recommenders) would provide information about the applicant’s background and thus possibly influence the admission committee’s perception of the candidate and preclude a “behind a screen” appraisal of the performance without extraneous and possibly misleading contextual clues about socioeconomic status. In addition, many, if not most applicants to liberal arts colleges do not yet know what they want to do, so it is not clear how the audition experience would be mimicked. 

We suggest that there are four ways of supplementing traditional admissions assessments that would reflect the emphasis of conservatories on the skills that really matter for future endeavors. First, one can require a major project in any area of endeavor that resonates with the applicant, and that shows what the applicant can do, including attained level of technical proficiency, in an area of interest to them. The idea is to assess their knowledge and skills in actually completing a project rather than merely in the abstract. Second, one can measure creative, practical, and wisdom-based skills in addition to the memory and analytical skills that are traditionally measured by standardized tests and grades in high school (Sternberg 2010, 2015). Third, one can measure more traditional analytical skills, but in discipline-relevant ways (e.g., Sternberg 2020a). Fourth, one can measure conative and motivation-related skills and traits. Any of these procedures would represent an enhancement of current practice. The problem with current practice is not only that it represents a too-narrow range of skills, but also that it sends the wrong message about what matters for future success, both in college and beyond the college years.

## 6. Conclusions

The conservatory model of undergraduate education has useful implications for how schools might rethink admissions, instruction, and assessment to further both domain-specific knowledge and more wide-ranging adaptive creative, analytical, practical, and wisdom-based intellectual skills. Educators often think of the conservatory as a place exclusively for technical training, but that is not what the modern conservatory is. Rather, it is a place to learn how to learn, learn how to think, learn how to adapt, and learn how to perform in real, consequential worldly settings. We suggest that, to the extent schools wish to re-envision education, in general, especially as education develops adaptive skills, conservatory training can teach educators as much as—or more than—it teaches the students who participate in it.

There are various ways of testing some of the ideas in this essay. First, one could test openness to experience with a typical performance measure and cognitive flexibility with a maximum performance at the beginning and end of college to determine how a liberal arts curriculum versus a conservatory curriculum impacts each, if at all. Some colleges, such as Oberlin, Ithaca, and Gettysburg Colleges, have both kinds of curricula, enabling something of a direct comparison. Second, one could survey human relations offices across diverse fields, including music, to survey their openness to hiring both liberal arts and conservatory graduates. The study even could be carried out as an experiment where hypothetical applicants are presented with identical credentials, except that each hypothetical applicant is presented either as a conservatory or a liberal arts graduate. The experimenter then could determine whether one kind of graduate is preferred over the other, independent of actual credentials. Third, one could compare professionally valued accomplishments of graduates of liberal arts and conservatory programs in a variety of professions, including, but not limited to, music. 

We believe, in the end, though, that the comparison between conservatory and liberal arts programs should not be viewed simply as a competition for which is better than the other. Rather, the two kinds of programs should be viewed as each offering the other features that can optimize the education they provide to their students. Each has unique advantages that, when thoughtfully combined, might create a truly outstanding instructional program at the undergraduate level.
